# Intersectional dynamics and care disparities in intrapartum electronic fetal monitoring: a socio-technical systems perspective

**DOI:** 10.1186/s12884-025-07765-z

**Published:** 2025-06-02

**Authors:** Verónica Blanco Gutiérrez, Lyuba V. Bozhilova, Natalie Darko, Antoniya Georgieva, Kenton O’Hara

**Affiliations:** 1 EPSRC Centre for Doctoral Training in Digital Health and Care, University of Bristol, Bristol, UK; 2 Oxford Labour Monitoring Group, Nuffield Department of Women’s & Reproductive Health, University of Oxford, Oxford, UK; 3 College of Life Sciences, University of Leicester, Leicester, UK; 4 NIHR Leicester Biomedical Research Centre, Leicester, UK; 5 School of Computer Science, University of Bristol, Merchant Venturers Building, Bristol, UK

**Keywords:** Qualitative research, Intrapartum electronic fetal monitoring, Social determinants of health, Decision-making, CTG, Perinatal disparities

## Abstract

**Background:**

Intrapartum Electronic Fetal Monitoring interpretation is subjective, variable and dependent on clinical expertise. Electronic Fetal Monitoring is also influenced by human factors, such as the labour ward context, staffing pressures, situational awareness, local protocols, workflow variations, team dynamics, and reporting cultures. This paper explored whether, and how, socio-technical factors may have the potential to contribute to disparities in intrapartum Electronic Fetal Monitoring care and their implications for maternal and neonatal health.

**Methods:**

This study employed an exploratory qualitative design to investigate clinicians’ experiences of Electronic Fetal Monitoring. Eighteen semi-structured interviews were undertaken online with midwives, student midwives and obstetricians involved in labour ward care in the UK. Critical Race Feminism and Intersectionality theories shaped the study design and analysis. Interviews were analysed using reflexive thematic analysis.

**Results:**

Seven themes were identified under the overarching theme intersectional dynamics in intrapartum Electronic Fetal Monitoring: 1) Social determinants in Electronic Fetal Monitoring interpretation, (2) Disparities in care expectations and decision agency, (3) Cultural influence on decision choices, (4) Disparities in communication, *(5)* Rationalising Electronic Fetal Monitoring outcomes towards preferred course of action, (6) Stereotypes and bias, and (7) Wider influences of Electronic Fetal Monitoring and labour care.

**Conclusions:**

Electronic Fetal Monitoring is a socially and contextually interpreted tool used to support particular interventions or inactions. Electronic Fetal Monitoring management is subject to systematic contextual influences, maternal Social Determinants of Health and biases that may further contribute to disparities in labour care and outcomes. Addressing maternal Social Determinants of Health while providing Electronic Fetal Monitoring care is vital to promoting equitable care, facilitating a positive experience and improving health outcomes.

## Introduction

Disparities in maternal and neonatal outcomes remain a pressing public health concern, particularly for women from minoritised ethnic groups and socioeconomically disadvantaged backgrounds [1–3]. Improving perinatal outcomes and reducing disparities has become a global priority [4, 5]. Efforts to improve birth outcomes have seen the widespread adoption of intrapartum Electronic Fetal Monitoring (EFM) technology [4, 5], enabling clinical teams to assess key indicators of fetal well-being during labour and provide an evidence basis for clinical decisions towards safer maternity care. However, the role of intrapartum EFM in improving neonatal outcomes is debated [6], and its use remains controversial [7]. It has been acknowledged that continuous EFM increases the risk for interventions compared with intermittent auscultation [5]. Poor EFM management^1^ is frequently cited as a source of preventable harm in maternity care [8], contributing to negligence costs [9] and iatrogenic effects, such as higher rates of unnecessary medical interventions, caesarean sections and instrumental deliveries [6].

In the UK, women are classified into “high risk” categories in the presence of fetal or maternal health issues, which usually warrants EFM during labour. The National Institute for Health and Care Excellence (NICE) antenatal care guideline defines a ‘high risk’ pregnancy when ‘the likelihood of an adverse outcome for the woman or the baby is greater than that of the normal population’ (National Institute for Health and Care Excellence, 2021b). Nevertheless, there are currently no national standards for risk assessing women in maternity care in the UK [10]. Pregnant women are not only deemed at risk based on clinical factors. Social aspects, such as women who had no or limited antenatal care, women from Black, Asian (excluding Chinese), and mixed ethnic family backgrounds, living in the most deprived areas, and with one or more complex social factors, are placed into high-risk categories due to “an increased risk of death and may need closer monitoring and additional support” [11]. Furthermore, minoritised ethnic women and women from low socio-economic backgrounds are over represented in high risk factors that require obstetric care and interventions during labour, such as EFM and induction. A recent cross-sectional survey of UK maternity units revealed that the most frequent indications for induction were social risk factors / late booking (23%), fetal growth restriction (85%), fetal macrosomia/large for dates (41%), intra-uterine death/ previous intrauterine death or poor outcome (54%), diabetes (85%), pregnancy induced hypertension / pre-eclampsia / hypertension (72%) [12]. Moreover, it has been identified that women with low educational level and those living in disadvantaged areas had a greater likelihood of being induced than women with higher qualifications and women in advantaged neighbourhoods [13].

Regarding the birth place, Asian women were more likely to give birth in obstetric units, explainable by cultural factors and clinicians’ biases [14]. Disadvantaged women are also subjected to worse obstetric outcomes compared to women from White and high socioeconomic backgrounds, such as higher rates of caesarean sections [15], higher mortality and morbidity risk [1–3], perinatal brain injury [16], stillbirths and neonatal mortality [2]. Women from disadvantaged backgrounds are also subjected to disparities in clinical care, such as micro-aggressions, disrespectful [17, 18] and substandard care [19], biases in care provision [1], exclusion from decision-making processes [20], and more likely to receive non-medically justified differential care during pregnancy [17, 18]. Furthermore, a growing body of evidence highlights the effect of women’s ethnicity^2^ and Social Determinants of Health (SDH)^3^, such as socioeconomic status (SES), on clinician-driven decision-making [23], while unconscious bias [24] and implicit and explicit biases negatively influence communication, patient-clinician interactions and engagement [25].

Addressing existing perinatal disparities and the effects of current EFM limitations on birth outcomes is challenging. Recent research [26] advocates for a shift in focus to improve EFM management by incorporating a systems approach and moving away from EFM training and interpretation alone. EFM should be regarded as a complex socio-technical system [27], where interpretation, escalation, decision making and collaborative response mobilisation may or may not occur. These practices are highly affected by human factors and the labour ward culture and environment [26, 28]. While previous research on the socio-technical aspects of EFM does not explicitly address health and care disparities^4^ [26, 28], it offers an important socio-technical lens to examine areas where implicit factors may lead to differential EFM management that exacerbate potential care disparities. The present study focuses primarily on EFM and potential sources of biases in EFM management in labour. Therefore, intermittent auscultation was not explicitly addressed in this research and it is beyond the scope of this study.

This paper explores whether, and how, socio-technical factors may may potentially contribute to disparities in intrapartum Electronic Fetal Monitoring care^3^ and their implications for maternal and neonatal health. A socio-technical systems approach recognises the human and emotional aspects of EFM management- and acknowledges that “no one comes to work to deliberately give sub-optimal care” [30]. This systems thinking shits away from individual blame cultures and focuses on recognising everyday challenges and pressures of care delivery in current healthcare systems [31]. By advancing this socio-technical systems understanding, we can promote curiosity and collective work towards a more equitable maternity care system that is set up to deliver evidence-based, culturally sensitive care for all women, regardless of their background.

## Methods

### Project background

Over the past decades, there have been efforts to address the variability in CTG interpretation with algorithmic support tools [32]. One such example is the Fit for Labour clinical decision support tool (CDST) prototype to predict risk of perinatal adverse outcomes [33, 34]. There is considerable interest in understanding how to extend algorithmic models to take into account and address perinatal health disparities across different socioeconomic and ethnic populations. In this regard, there is further interest on the potential role that socioeconomic and ethnic factors may play in larger socio-technical system of EFM care, such as escalation, response practices, collaborative decision making, situational awareness, team power dynamics and workflows in which such algorithmic intrapartum decision support tools are embedded. With this in mind, the primary author is carrying a doctoral research to combine data-centric framings with socio-technical perspective to help CDST model development and implementation in order to mitigate health inequalities in intrapartum care.

The study reported in this paper is part of a broader set of studies. A Patient and Public Involvement and Engagement (PPIE) group of minoritised ethnic women was set up at the start of the doctoral research to provide critical feedback on the project, identify unmet needs and inform future research. This PPIE project partnered with community organisations supporting minoritised ethnic women. Art-based methods were used to build trust and facilitate different ways of expressing themselves, particularly when discussing sensitive topics. A manuscript on this project is under development and will be published in the near future. While logistical challenges delayed the formal launch of the PPIE project until after the qualitative study had commenced, insights from one-to-one meetings with women prompted refinements to the interview questions used in the current study with clinicians.

### Positionality statement

VBG is a registered midwife with extensive clinical experience in labour ward and self-identifies as a White ethnic minority. Inevitably, past personal and clinical experiences have likely shaped how the researchers interpreted the participants’ experiences and understanding of the maternity and labour ward contexts, the interactions between patient-clinician, racist and discriminatory encounters, and sources of biases in maternity care. LVB is a Senior Postdoctoral Researcher in Maternity Data Analysis & Fetal Risk Assessment. ND is an Associate Professor of Health Inequalities and Director of Inclusion at the Leicester National Institute of Health Research Biomedical Research Centre. AG is an Associate Professor, Group Lead - Oxford Labour Monitoring. KO is a Professor in Human-Computer Interaction, specialising in health care contexts.

### Methodology: critical race feminism and intersectionality theories

Critical Race Feminism (CRF) combines the principles of Critical Race Theory (CRT) and feminism, resulting in a “race critique within feminist discourse” and “a feminist critique within Critical Race Theory (Wing, 2007). CRF places women of colour^5^ at the center [36], and acknowledges that race/ethnicity are socially constructed categories [37], racialised women are seen as the “others” [38] and stereotypes homogenise women by reducing diversity and individual experiences [39]. Incorporating an intersectionality framework is vital to understand the intersection of multiple identities, such as socio-economic status, race/ethnicity, gender, religion, language, education, nationality [39], and how they contribute to varying levels of privilege and oppression [40].

CRF and Intersectionality frameworks have been used in maternal health research to address systemic and structural bias [41–43]. Therefore, both frameworks were selected as they provide an adequate analytical lens for this research. CRF and Intersectionality theories have shaped this research, both in the study design and the analysis of the findings.

### Study design

Semi-structured qualitative interviews were used to understand midwife and obstetrician perspectives on intrapartum EFM management and care. The study aimed to explore whether and how the sociotechnical context of EFM management may have manifested in care disparities among women from disadvantaged groups. Approval to conduct the study was sought from the University of Bristol Ethics Committee (Ref. 16538).

### Participants

Participants were selected using convenience sampling. The study was advertised between January and May 2024 on Social Media platforms and professional networks, such as LinkedIn, Facebook, Twitter/X, JICSM@ail and word of mouth. A study flyer and a website were distributed containing information about the study, participant information sheet, eligibility criteria and how to take part. Criteria for selection included midwives, student midwives and obstetricians (including doctors in specialist training and consultant obstetricians) involved in labour ward care in the last two years in the United Kingdom (UK), with access to the Internet and electronic devices, and the ability to communicate in English.

Demographic data were captured in a brief, anonymised questionnaire (Table 1). Fifteen clinicians (thirteen midwives, two obstetricians) and three student midwives met the criteria and participated in the interviews. Participants were given a participant information sheet for further details about the study and completed an electronic consent form following an opportunity to ask further questions.

**Table 1:** Participants’ demographics

Role	Age	Gender	Ethnicity	English as first language	County of training	Education	Clinical experience	Location
Midwife	36	Woman	White	Yes	Outside UK	Degree	> 10 years	Grater London (England)
Midwife	42	Woman	White	Yes	UK	Masters	< 1 year	Scotland
Midwife	38	Woman	Black	No	UK	Masters	6–10 years	South East (England)
Midwife	34	Woman	White	Yes	UK	Degree	1–5 years	Midlands (England)
Midwife	34	Woman	White	Yes	UK	Degree	6–10 years	South East (England)
Midwife	23	Woman	Asian	Yes	UK	Masters	1–5 years	Greater London (England)
Midwife	33	Woman	White	No	Outside UK	PhD	> 10 years	Greater London (England)
Student	42	Woman	White	Yes	UK	Degree	2nd year	South East (England)
Student	23	Woman	White	Yes	UK	Degree	3rd year	South West (England)
Midwife	32	Woman	White	Yes	UK	Degree	> 10 years	North (England)
Obstetrician	41	Woman	White	No	UK	Degree	> 10 years	Wales
Student	19	Woman	White	Yes	UK	Degree	2nd year	South West (England)
Midwife	44	Woman	White	Yes	UK	Degree	> 10 years	North West (England)
Midwife	34	Woman	White	Yes	UK	Degree	6–9 years	South West (England)
Midwife	28	Woman	White	Yes	UK	Degree	6–9 years	Greater London (England)
Midwife	44	Woman	Black	Yes	UK	Degree	> 10 years	Greater London (England)
Midwife Obstetrician	34 -	Woman Man	White -	Yes -	UK -	Degree -	8 years -	North West (England) Greater London (England)

During the interview, participants provided information about the local context in which they practiced. Participants worked in a mixture of small, medium and large size hospitals, including big-city hospitals and university hospitals. Most of the participants worked in high-risk obstetric units although some midwives provided a mixture of low and high-risk care. Regarding clinicians’ demographics, the majority of participants reported that their demographics did not match those of the populations they looked after. Exposure to diverse ethnic and socio-economic populations also varied amongst participants; in some hospitals, ethnic minoritised women and women from low-socioeconomic backgrounds represented the majority while in others the majority of the service users were from White backgrounds. In regards to resources, the availability and usage of EFM technology (telemetry, ST-Analyser, central monitoring) and translation and interpretation services varied across hospitals. Different interpretation guidelines were used by different maternity services, including FIGO, NICE, physiological interpretation and a combination of some of the guidelines mentioned (i.e. NICE and physiological interpretation). In some labour wards, multiple guidelines coexisted, consciously or unconsciously, as a result of unit’s preferences, staff rotations and relocation. The delivery of culturally sensitive and human factors training also varied across units. Participants’ views and sentiments on maternity care disparities and issues in the current provision of maternity care in the NHS were fairly heterogeneous.

### Data collection

The interviews were conducted between January and May 2024 via Microsoft Teams. Interviews ranged from 50 to 120 min and contained open-ended questions and vignettes to explore participants’ experiences of intrapartum EFM monitoring and care practices. The vignettes used to support the interviews were based on the experiences of Black, Brown and Mixed-ethnicity women collected in the Birthrights report [19]. The scenarios were carefully selected to represent well-documented stereotypes in maternity care, particularly those stereotypes that may unconsciously influence clinicians’ behaviour. This indirect approach provided a safe framework for participants to reflect on systemic biases and explore their potential impact on electronic fetal monitoring without focusing on personal practices.

Vignettes are used in qualitative research to trigger the participant’s reflection about their feelings or actions in that context. Their use is advantageous when researching sensitive topics since it encourages participants to share their personal experiences and beliefs without being the focus of attention [44–46]. Tables 2 and 3 show the interview guide and examples of vignette scenarios and themes that they sought to address. All interviews were recorded and transcribed with the embedded Teams transcription option and reviewed and corrected to ensure accuracy. Data saturation was achieved when the research team reached a consensus that no new information was emerging from the interviews. Participants were thanked and compensated for their time with a £25 gift card.

**Table 2:** Interview guide

**Introduction** - Tell me about you, your role, experience and place of work.
**EFM and decision-making** - What are your thoughts on EFM for labour monitoring and its role in decision-making? - Reflecting on a recent case: what non-clinical factors influence EFM management?
**Personal characteristics** - What are the characteristics of the workforce and population that you look after? - How do you think SDH influence perinatal health? - Reflecting on sources of perinatal health inequalities and encounters with discrimination and racism.
**Vignette-based questions** - Reflecting on stereotypes based on ethnicity, physical attributes and language barriers. - Influence of maternal characteristics (ethnicity, language, origin, age, etc.) on informed decisions, consent and choice in the context of labour care, specifically on EFM.

**Table 3:** Examples of vignette scenarios and themes addressed

Vignette scenarios	Themes
**Scenario 1** Thinking about an episode where you looked after a woman in labour in the past 2 weeks (clinical context: high risk labour with EFM). Please walk me through the contextual factors/non clinical factors or events that you might be interested in to develop the clinical picture for the woman and the baby.	Reflecting on non-clinical factors that influence EFM management.
**Scenario 2** An Indian woman overhears a conversation between two midwives about how “Asian ladies are too precious” and do not tolerate vaginal examinations very well. “They always make a fuss”.	Microaggressions, racist attitudes and behaviours manifesting as stereotypes and their influence on respectful labour care.
**Scenario 3** An Arab woman wearing a head scarf is in labour. The clinician speaks slowly, with simple terms and from a distance. This woman then says that she is a pharmacist and suddenly the doctor changes the way he speaks to her.	Stereotypes and attitudes based on physical attributes/characteristics in the context of communication during labour. Respected professions provide benefits and power that influence how care is provided.
**Scenario 4** A Black woman is in labour. At some point, her contractions stop and on the next vaginal examination she is diagnosed with slow progress. The midwife then explains the woman about the “African pelvis” and tells her that they need to start her on the hormone drip. The midwife gets everything ready and starts the infusion without discussing pain relief options with the woman as she thinks that Black women have very high tolerance to pain.	The use of bias and stereotypes to inform clinical judgement.
**Scenario 5** A woman who is not fluent in English is in labour. The CTG assessment shows signs of fetal distress, but it does not require an urgent intervention at the time. The midwife has noted that the woman has difficulties to understand what is communicated to her. Instead of offering some “extra” time, the doctor makes the call for a Category 3 cesarean section (non-urgent caesarean section but early delivery is required).	Language barriers and their influence on clinical decision making and women’s choice and autonomy during labour.

### Data analysis

Interview transcripts were organised using NVIVO 14 software and Microsoft Excel. A reflexive thematic analysis approach (RTA) [47] was followed, both inductive and deductive, in order to capture and take into account the lead researcher’s subjectivity as a midwife [48]. The researchers conducted the analysis following the RTA steps. Firstly, the researchers familiarised themselves with the data by listening to the audio recordings and reading the transcriptions several times. In the first rounds, notes were taken, and tentative codes were noted. After the familiarisation stage, initial codes were generated. Codes were reviewed with the research team. Final codes were chosen, and discrepancies were resolved. With subsequent iterations, codes were categorised into meaningful themes and relevant quotes were identified.

## Findings

**Fig. 1:**
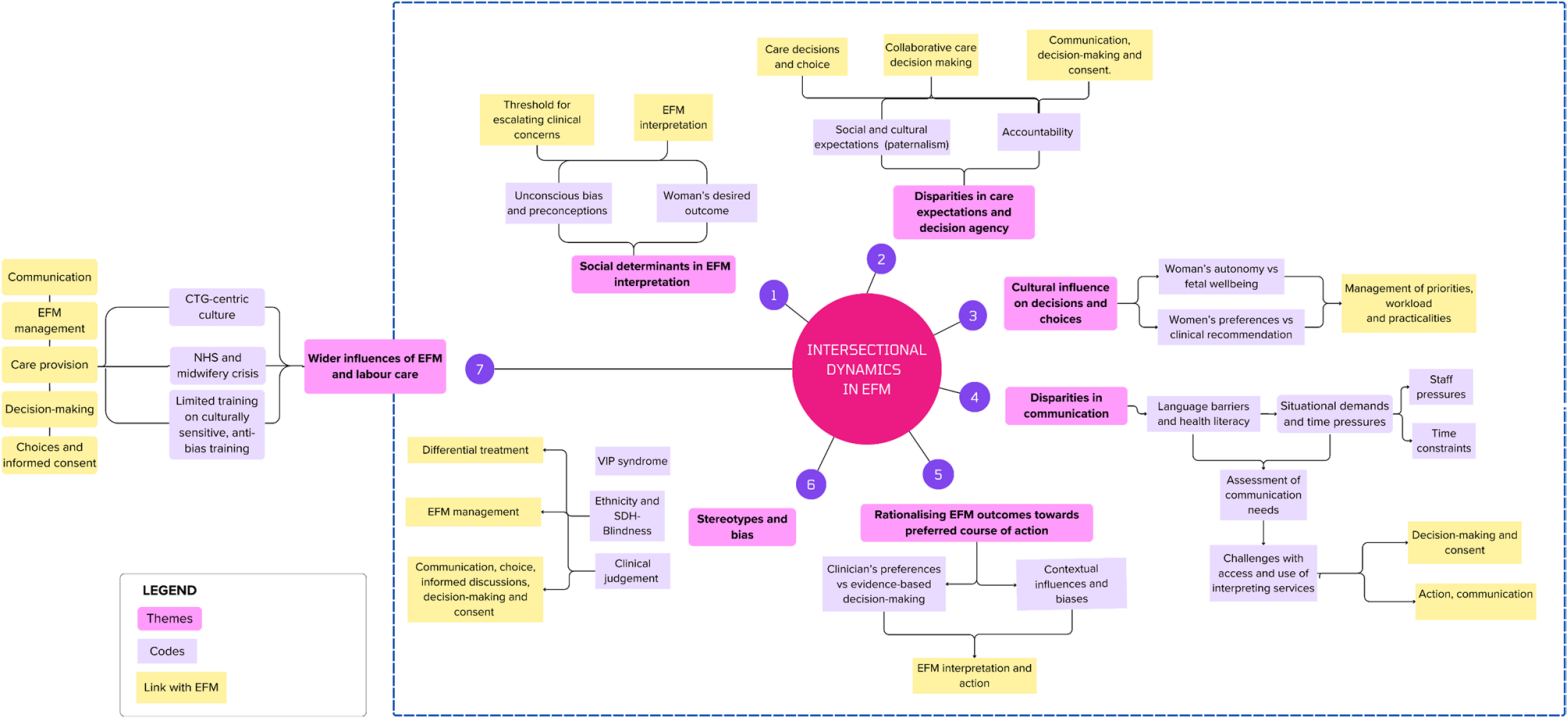
Intersectional dynamics in EFM management from a socio-technical perspective

Seven themes were evident from the interviews under the overarching theme intersectional dynamics in intrapartum EFM (see Fig. 1). Intersectional dynamics refers to the overlap between women’s and clinicians’ preferences and recommendations, physical attributes and SDH, and how they played a role and may have potentially influenced clinical interactions. The intersectional dynamics were evident in several areas, such as the role of SDH in EFM interpretation, expectations and decision agency, maternal choices and cultural influences, communication disparities, preferred course of action and stereotypes and bias. Moreover, intersectional dynamics were impacted by wider influences of EFM and labour care.

Table 1 illustrates the characteristics of the participants. Reporting participants’ demographics is key to understanding the diversity of the participants’ perspectives on the topic and provides additional context to the findings. Collection of demographic data also serves to evaluate the representativeness of the sample and reflect on the study design and recruitment practices [49].

### Theme 1: social determinants in EFM interpretation

While participants acknowledged the influence of SDH on maternal and fetal wellbeing, they did not explicitly consider this in their readings and interpretation of the EFM trace. Rather, the visual representation of the EFM data, i.e. fetal heart rate and contractions, was considered independent from non-clinical factors.

*“Of course they would affect how the pregnancy goes*,* but in terms of the interpretation of the CTG*^6^*in itself*,* I don’t… I’m not sure we should be thinking about those things when we’re interpreting it. Like*,* of course*,* when we provide holistic care*,* we know the woman that we have and we know if she has any kind of worries or concerns or mental health or all these kind of things. But I’m not sure we should consider the country of birth or the immigration status or income*,* when we’ve considered interpretation of the CTG*,* that is more of the holistic care that you would provide in pregnancy kind of thing.” P8*,* Midwife.*

*“The interpretation of the CTG is never looked at in terms of her ethnicity or language barrier. We purely look at CTG based on CTG and the clinical background. So no*,* I don’t think all those kind of social implications or*,* you know*,* backgrounds and so that has an impact on our decision making around CTG interpretation”. P7*,* Midwife.*

In this regard, factors relating to SDH were not considered as something that should be included explicitly in interpretive guidelines or algorithms for classifying EFM outputs and intrapartum risk. Other clinicians, however, recognised that the threshold of their clinical judgment, for example, during escalation of EFM concerns, was influenced by the overall clinical picture, including SDH and other non-clinical factors that could place the woman and the unborn child in a vulnerable position.

*“I think it’s really important that people are understanding. So I’ve started bringing it into my teaching*,* ethnicity and saying like to this mother is social deprivation score one and she’s black African*,* so she’s got these… This is an extra vulnerability that you need to be aware of. You need to be thinking about the fact that she is more likely to get a poor outcome. So that needs to be in your mind because I don’t think we understand why we have a high rate of*,* like*,* these women of dying and… You know*,* if their babies dying*,* I don’t think we know why.*

*But it’s got to be in my mind*,* like racial bias. It’s just got to be that. But so I think we need to try make everyone aware at the very least*,* I don’t know how else we can kind of start to fix that problem. We’ve got a lot of lot to fix I think but with at least making everyone aware of*,* this woman is more vulnerable because of this*,* so we need to*,* you know*,* make sure that we’re aware of that. I think it may start to help”. P5*,* Midwife.*

Often, these SDH had more weight on clinical management when extreme social and safeguarding issues occurred. Some participants acknowledged several ways in which unconscious bias and preconceived notions may influence the interpretation of EFM readings, potentially shaping clinical decision-making and contributing to disparities in care.

*“But in terms of CTG interpretation*,* like you say*,* you presume that*,* younger women labour quicker or you presume that certain ethnicities don’t need pain relief*,* and maybe you do go into it with a little bit of judgement so that when it comes to interpreting*,* well*,* young women labour quickly. But actually*,* how can we presume young women labour quickly? We don’t*,* but you might go “Oh*,* that CTG is normal because she must be pushing”*,* but actually*,* she might not be even fully*,* and therefore it’s not normal*,* because that baby shouldn’t be that distressed when they’re in early labour. Do you know what I mean?” P16*,* Midwife.*

Participants also discussed how woman’s desired outcomes might unconsciously influence EFM interpretation and decisions.

*“If a woman is very keen on a vaginal birth*,* you might look at the positive things in an abnormal CTG*,* because*,* you see*,* is your assumption that this patient needs to give birth vaginally so therefore you would try and push things more than you may do for another individual who is not so*,* you know*,* bent on having vaginal birth”. P15*,* Obstetrician.*

*“If they are on the side of section anyway*,* maybe we’ll be more inclined to interpret the CTG as not reassuring or suspicious if they were sort of keen to have a caesarean section anyway.” P13*,* Midwife.*

### Theme 2: disparities in care expectations and decision agency

While shared decision-making about care during labour is a fundamental aspect of the UK woman-centred birth model, participants highlighted potential social and cultural disparities in expectations about maternal agency in care decisions in EFM management. Participants described how women from other countries and cultures, especially outside of Europe, were less inclined to enact any choice in their care decisions. Instead, there was a greater prevalence of a more “*paternalistic attitude*” (P15 obstetrician) to care decisions with greater deference to the doctors as decision-makers.

*“[…] whereas over here*,* it is very much… all management decisions are very much a dialogue between the doctor and the patient or the midwife and the patient or woman*,* whatever birthing person*,* you should say. Whereas I think in a lot of countries abroad*,* especially when you go more outside of Europe*,* it’s very much the doctor tells you what to do. And that’s what they are used to*,* and they’re not used to women asking questions or you actually explaining to a woman that there are two or three options and it is her choice which option to go for*,* you know. So yes*,* you do definitely see that” P6 obstetrician.*

The offer to participate in care choices was perceived to be a source of confusion for certain women from different countries and cultures, with persistent efforts towards shared decision-making sometimes making women uncomfortable. This was not simply regarded as deference towards the knowledge of healthcare professionals but was also bound up in expectations about accountability for their baby’s well-being– something that women preferred trained professionals to take responsibility for.

*“The more choice you give*,* the more people who are recent to the UK*,* they’re just like good naturedly*,* like*,* bemused. The more choices you offer them*,* they find it almost funny*,* and then they will just say*,* well*,* what should we do or the doctor will tell us what to do. And you’re like*,* OK*,* like*,* you know*,* you keep trying*,* you keep pushing them and they’re to a certain extent*,* you realise*,* I think I have to realise that*,* like*,* you’re making them uncomfortable. Because they would never be like expected to be held to account for their baby’s well-being*,* it is like you are the doctor*,* you’re a trained medic*,* like you’re a professional*,* you are in charge” P12*,* Midwife.*

Accompanying such deference and unquestioning delegation of responsibility was the perception that ethnic minoritised women would be less likely to engage with the whole process of collaborative care decision-making, speaking up less and asking fewer questions in the intrapartum and entire perinatal period.

*“I feel they don’t ask as many questions… They never question what we offer in terms of… choice. I definitely feel like they… a lot of these women never question our decision making” P9*,* Student Midwife.*

As a result, communication and engagement in the decision-making and consent processes were perceived and enacted differently in comparison to predominantly White, well-educated and articulated women with higher expectations and greatest awareness of maternity services. Moreover, communication and language barriers, logistic issues with translation services, time-constrains, workload and clinical priorities may have contributed to clinician’s gatekeeping attitudes to access and use translation services.

*“Yeah*,* and the discussions are a lot shorter and the consent process is a lot less thorough than if you’re talking to Sophie Roberts*,* it is completely different and so… If the person calls a cat 3 [category 3 caesarean section*,* non-urgent]*,* you’re not calling it a two*,* so you have time. Either you*,* the consultant*,* the registrar*,* get the interpreter services and go through it with this woman because it’s major surgery and something can go wrong and the woman can say ”I was not fully aware of what I was putting myself through”*,* and all the consequences or the potential risk of this surgery” P4*,* Midwife.*

### Theme 3: cultural influence on decisions and choices

Culture was perceived as having a strong influence on some women’s choices and decisions during labour and how EFM findings were managed and acted upon. Women’s preferences were sometimes based on cultural beliefs and practices which could often clash with clinical recommendations.

*“Sometimes it will be stressful because they [Orthodox Jewish] won’t sign a consent form for going for a category one caesarean and again*,* like you can say… Well*,* we really need to do this now. And so then that can cause a lot of stress*,*… and then again the way around it is you just take verbal consent because*,* what can you do? But sometimes there’ll be delays as well with different cultures. If they want to speak a religious leader*,* and they might want to run the decision past them*,* and in some religions*,* they might decide that a baby dying is the best thing at that moment*,* and that’s very hard*,* you know*,* you’re watching. So it’s just different in every culture*,*… a lot of the Tigrinya women think that a caesarean section is really dangerous*,* so because of that we’ve got quite a number of Tigrinya women that will decline caesarean sections and then you’re watching their baby on the CTG*,* getting more and more hypoxic*,* and you’re just thinking… We could stop this so*,* but you have to respect the wishes of the woman… but you’re also trying to be an advocate for them.… It’s very challenging” P5*,* Midwife*.

In cases where women’s autonomy is prioritised over fetal wellbeing, it was felt to be frustrating and challenging for the clinical team to understand. Furthermore, additional practicalities and processes of accommodating cultural needs were seen as something that would add work and delay in already resource-stretched contexts. This additional workload can contribute to pressures to shortcut and deprioritise some concerns.

### Theme 4: disparities in communication

Communicating EFM findings and explaining the proposed course of action to women and their birth partners was seen as a key aspect of EFM management and shared decision-making. Communication and language barriers were frequently reported to present particular challenges to shared decision-making and consent practices in the context of clinical response to particular traces, creating a potential source of care inequalities. While it was acknowledged that women experiencing language barriers should have an interpreter throughout their labour, it was also recognised that such services also incur logistical and practical challenges that take time and effort to enact in situ. In consequence, it has been normalised for translation service options to be more discretionary, and more likely to be restricted to circumstances that a clinician deems important.

*“No*,* not enough. We don’t tend to use it for labour. I think we get by in labour care…If we have time and it is failure to progress or it’s maternal choice and definitely then we can use language line and you know*,* it’s interpreted over the phone. But I don’t think it’s used commonly enough”. P3*,* Midwife.*

Assessment of woman’s communication needs was regarded as subjective and dependent on the broader context of the ward. It was felt that there was often a convenience of over-relying on the use of family, friends and students as translators and communicators. However, there was some critique of the assumption that they can do so sufficiently, placing an undue burden on them and failing to acknowledge the difference between health literacy and language barriers.

*“[when] you need to make a decision*,* so you want to make sure that there is more understanding of what the situation is and allowing that extra time but you can’t have a language line on [translation services] for the whole duration of labour*,* but that woman should really be able to communicate with the people that are looking after her throughout labour. When we rely just on the partner*,* again*,* we’re making an assumption that he’s going to translate the right things at the right time and he’s not going to coerce her or manipulate her decision in any way*,* which is really wrong… So yeah*,* we’re not doing the right thing with these poor women in that sense”. P8*,* Midwife.*

For some participants, language barriers were perceived as “inconvenient” language barriers and were seen to lead to dismissive behaviours by certain healthcare staff, excluding women facing communication challenges from the shared decision-making:

*“And then in the brief he said “I’m not having language line for the section”*,* he said they weren’t that good and it was just painful to speak to them on the phone and I just thought*,* like*,* come on*,* like*,* she is probably terrified*,* she speaks no English*,* she can’t tell you what she’s feeling like. How can you be so dismissive of someone? So just things like that*,* like the care kind of differs” P9*,* Student Midwife.*

In spite of good intentions, situational circumstances compromised challenging and time-consuming translation-mediated communications. For example, staff pressures in under-resourced maternity units, compounded by acute emergency demands across other women on the unit, meant that translation efforts would be sacrificed.

*“I know that has happened*,* whereby they just couldn’t get a hold of language line and again*,* I think external factors contributed to the decision making*,* especially like on nights and then it’s*,* you know*,* cat one [immediate threat to the life of the woman or fetus.] and then cat two [maternal or fetal compromise*,* which is not immediately life threatening] and then you’re just like*,* “OK*,* how best do I manage?” You know*,* what’s going on. And then there’s a PPH [Postpartum haemorrhage] in one room*,* so I think*,* for me*,* in terms of experience*,* I think it’s been a case of external factors that have kind of pushed for that [not using Language Line]… Some doctors*,* not all doctors… they don’t recognise that is just as important that communicate your decision making and sometimes*,* you do find that if it is a horrendous night*,* you can understand the thought processes of why this does happen. Not saying it should happen*,* but you get it You do have to make those clinical decisions”. P18*,* Midwife.*

The consequent lack of effective communication with women during labour led to disparities in care and their participation in shared decision-making. Participants felt that some women received different and less accurate information, depending on their level of understanding and their perceived socio-demographic characteristics, leaving women unable to fully understand the clinical situation and the information presented.

*“[…] I think a lot of midwives they don’t want to spend the time counselling them [women who might lack education around labour] properly and explain to them the risks and the benefits of every type of analgesia and they’ll just go straight to you know… Oh*,* I think it’s time for an epidural and it just saves them time in their opinion”. P2*,* Midwife.*

The challenge of language barriers also influenced beyond the immediate practicalities of communication. Anticipatory measures to address expected language barriers, such as initiating clinical interventions earlier than medically indicated to accommodate translation or interpretation, may have influenced clinicians’ decisions when responding to EFM readings. This scenario could point to a potential source of care disparities originated from a communication disparities standpoint across women experiencing language barriers.

*“I’ve heard of people [clinicians] moving people [women in labour] to theatre sooner to allow for more time because*,* you know*,* of the interpretation stuff*,* so if someone’s like*,* you know*,* got a dodgy CTG and they’re like 9 centimetres*,* they’re more likely to call for a trial [attempting a vaginal delivery with instruments before undertaking a caesarean section] beforehand when they get to anterior lip*,* they’re going to get them into theatre because they’re anticipating it’s going to be*,* maybe a difficult delivery in terms of communication. And you need more time to prep*,* and that kind of stuff*,* but not like literally the reason for doing a section is because they don’t speak much English*,* like obviously*,* on their op [operative] note*,* they’ll put reason for section fetal distress*,* right?” P12*,* Midwife.*

### Theme 5: rationalising EFM outcomes towards preferred course of action

Interpreting EFM traces and associated decision-making were seen as subject to clinical judgment on the part of the care team. The interpretive flexibility and plausible ambiguity of EFM assessments offered scope for trace readings to be invoked as a means to rationalise and justify preferred courses of action for the clinician– as opposed to being the basis of a more collaborative approach to decision-making.

*“I don’t think we’re led by the CTG*,* I think you get a kind of a vibe and a clinician*,* a senior doctor might have an idea of what they want to do*,* and then that is the lead point of the decision making and the CTG feeds into that. I think sometimes people make CTG what they want to be unless it’s really obviously normal or really obviously pathological…I think in that middle ground you can make a CTG what you want as to what you want to do for your decision making*,* which is not how it’s supposed to be*,* but that’s how it is I feel”.P12*,* Midwife.*

*“I think it’s used as a bit of a tool to get people to do what the obstetrician wants*,* which is lie in a bed and be monitored.” P13*,* Midwife.*

### Theme 6: stereotypes and bias

Some participants indicated that prior experience of labour care for women from different cultures and socioeconomic backgrounds sometimes manifested in clustered characterisations of labour behaviours based on maternal characteristics, such as age, SES, ethnicity, religion, culture, country of birth and health literacy– whereby women with similar demographics were seen to experience labour in a common way. These stereotypical belief systems were felt to subsequently influence clinical decision-making and EFM management.

*“I might say I’ve got a patient who’s a black African woman and she’s not really dilating very quickly*,* but in my mind*,* I’ll be thinking that I think she’d hit second stage quite quickly because that’s something that women do. I’m not saying it is a negative sort of way at all*,* I’m just using my experience. I think she might labour this way”. P13*,* Midwife.*

Others reported witnessing how preconceptions about women, based on looks and socioeconomic factors, could have influenced the ways that clinicians communicated and interacted with them. For example, assumptions about low education levels and health literacy of women affected whether explanations for clinical actions would be given and even limited the care choices offered to them.

*“It’s just assuming that you’re not gonna understand and talking to them as though they’re stupid*,* really. And it’s like you*,* you literally could be a cardiologist and just based on your looks”. P11*,* Student Midwife.*

There is a biased assumption here of limited capacity to understand the choices and explanations offered, resulting in exclusionary behaviours simply based on stereotyping. Maternal preferences are sometimes assumed or ignored. Time demands and heavy workloads could further exacerbate exclusionary behaviours and assumptions as clinicians become pressurised into employing shortcuts in their decision-making, and therefore, potentially more prone to unconscious bias in their judgment.

There was also a suggestion of where such biases played out in disparities in patient-activated escalation creating barriers to self-advocacy during labour. For example, this could affect the clinical interpretation of self-advocacy behaviours such as expressions of pain or concern. Judgments about the seriousness of complaints in turn could affect the level and speed of response– leading to delays in care based on stereotypical assumptions.

*“I think they are very delayed in acting on things when you are from an ethnic minority*,* if the woman doesn’t speak English*,* it’s likely to not to complain much. If you’re Black*,* for example*,* and you say something*,* you make the stereotype maybe*,* oh*,* she’s a troublemaker. So therefore*,* you asked for help*,* they take forever to come to you with help. If you were called Hannah Smith*,* the help comes quickly. If you are called Hannah Smith and on top of that*,* you are a lawyer*,* the midwife will walk you to the bed and do whatever you ask her to do. And if you are call Hannah Smith double barrel Johnson*,* the consultant will come and talk to you.” P4*,* Midwife.*

*“So these women feel like they’re not listened to*,* they feel like their pain isn’t taken seriously. They feel like they’re not valued*,* like if they complain*,* they’re seen as being a nuisance.” P12*,* Midwife.*

Some participants, by contrast, acknowledged care disparities but in a different direction. Being aware of current perinatal disparities, participants suggested their tendency to overcompensate in their care behaviours towards perceived vulnerable women, i.e. refugees, women with limited English and women with low SES. Such behaviours were viewed as an attempt to mitigate the social injustice:

*“I think sometimes*,* if anything*,* I try and favour people with a poorer background or refugees or something*,* […] I’m very aware of the things that they face in the NHS and I’m always sort of an advocate for them*,* even though they don’t really know that I am. So when women come from a poor background*,* I don’t think of things like that… I think of more how can I improve your experience. In terms of*,* what do you need? Do you need language line? Do you need things like that? Because I’ve seen it before*,* where women who don’t have English as their first language or come from a poor background aren’t really given that same care and it impacts on health outcomes. ” P9*,* Student Midwife.*

*Sadly*,* I think it does have a big impact on women’s health [perinatal disparities]. Unfortunately*,* like it’s very well documented that women of*,* I think*,* minorities are more likely to die or experience trauma and injury during their during their labour and pregnancy and birth. So aware of it or not*,* it is happening. And that is true also if people have more deprived socio economic backgrounds. It’s a fact*,* It doesn’t matter if I agree with it or not*,* it’s still happening*,* it’s proven that it does impact the care. I tell my students like*,* especially when we go around*,* they’ll ask why did you spend more time with this person than the other person? I say although I’ll never intentionally be biassed in the care I give*,* I’ve got this awareness that women are receiving poorer care. They’re having poor outcomes from these specific backgrounds. So I find it important to spend a little bit more time with them*,* when time allows*,* just to really feel like I’ve thoroughly ticked all my boxes and understand that… I think a lot of the time*,* there can be that culture*,* they don’t really want to ask for help*,* so just making sure that they’re fully aware I am here*,* you’re not wasting my time*,* whatever you need*,* just ask me. And then spending a little bit more time with them can… well I think hopefully will make it more likely they’ll ask for help if needed. And they’ll try and relate that to my students as well.” P14*,* Midwife.*

Consensus about perinatal inequalities was not always apparent, with numerous participants also highlighting their belief that they care for all of their mothers the same, irrespective of their perceived background.

*“It doesn’t matter what they’re wearing*,* you know*,* they could wear a tracksuit and be a millionaire. You don’t know*,* do you? And you don’t really care… I’ve never met that lady before*,* I go in and she is six centimetres*,* I’ll just look after her in labour like anybody else […] level of education? No*,* probably not. I don’t know the level of education when they’re in labour*,* so no*,* I don’t really need to know the level of education ‘cause I’m looking after all women the same… particularly*,* all their babies are the same”. P13*,* Midwife.*

While such a perspective may seek to avoid any explicit bias, it may also exhibit “blindness” to the potential need for personalised care that can accommodate SDH factors in care.

### Theme 7: wider influences of EFM and labour care

During the interviews, broader factors influencing EFM and labour care emerged. Participants highlighted a CTG-centric culture on labour ward, where EFM was regarded as a key intervention for high risk women. Some participants reflected on the overuse of EFM without clinical indication to comply with the local culture.

*I think it’s important*,* especially when you’ve got a high risk woman or high risk baby. But I think sometimes*,* we’re all guilty of over CTG-ing women. Sometimes you do it and you’re questioning why are we actually doing this? But it’s just because that’s what we do here and especially having worked in different trusts*,* when I came to [different hospital] it was like oh*,* she needs a CTG and it was a bit like but why?… it’s the culture of like*,* let’s just do it…” P16*,* Midwife.*

An additional influence of EFM was the national maternity landscape and the impact on the workforce. Staff shortages were an shared theme across participants. This landscape highlighted the unrealistic workload that midwives and doctors were required to do, which ultimately could have impacted on the care provided, particularly around choice and autonomy. In this climate of tension, frustration and dissatisfaction with the care provided were common, as clinicians, particularly midwives, felt that they had to act against their values. Labour care was seen as reactive and reduced to addressing what participants considered the “basics”, where choice and autonomy were regarded as a luxury.

*“I think*,* because we’re in such a crisis with midwives*,* I think what we want to do and what we end up doing are two different things. I think technically*,* in terms of policies and procedures that you have at the hospital*,* I think it kind of goes with what we want the end result to be like*,* you know*,* follow the guidelines and then give that woman every possible chance to kind of go into labour naturally. And then everybody has like the birth that they wanted or can have. But I think having not so many midwives does play a part in how we manage who we’ve got on the board*,* which is sad but I can understand that […].It hurts… the way that midwives in the system are at the moment*,* it’s almost being not fit for purpose”. P18*,* Midwife.*

Maternity investigations and the negative press about the NHS were reported to have a negative impact on clinicians’ morale. Clinicians felt frustrated and caught in a system that promotes choice and autonomy but fails to address how human factors- time, pressure, stress, workload- shape the organisational culture, interactions, behaviours and clinical practice.

*“[…] I think the public*,* the population*,* I think they really forget that we don’t go to work to be horrible people. You know*,* we are there to do our best and to make the best experience for them. But sometimes these things are also out of our control and we just have to make the best of it. But I think that’s just the way public opinion is going at the moment*,* isn’t it?”. P6*,* Obstetrician.*

Clinicians’ morale appeared to be undermined by solely focusing on individual-level errors and promoting a blaming culture. Moreover, the negative press about maternity services was identified to influencing women’s trust in clinicians and moulding women’s childbirth preferences.

“*And I think we’ve had a lot of reports lately*,* like especially with the EMBRACE reports and the different things that are coming out about […] like the Ockenden and things like that*,* a lot of them have highlighted that we don’t interpret CTGs perfect every time despite the best way we try”. P3*,* Midwife.*

*“I think it’s very variable. We have some people who want you to make decisions for them and some people who very much want to make their own decisions. I think most people do follow recommendations for the most part*,* but you know*,* choices become apparent now*,* all the maternity reports in recent years*,* doesn’t paint a very good picture of maternity care and I think there’s lack of trust*,* particularly with vaginal birth*,* induction of labours. If you look at the maternity reports*,* they’ve talked about judicious use of oxytocin*,* people don’t want to be induced these days. So definitely our numbers of women asking for maternal request caesareans have definitely gone up. People want to have a caesarean section as opposed to having an induction”. P15*,* Obstetrician.*

Participants reported limited or no training on culturally sensitive care, bias and anti-racism specific to maternity. Training lacked meaningful and profound change and was seen as a mere tick box exercise. Moreover, the political scene was perceived to influence people’s views and behaviours around bias and racism, including clinicians. Consequently, cultural transformation was understood as an accessory in times of hardship.

*“[…] there’s still a lot of subconscious and unconscious bias among clinicians*,* and I always get cautious when people are so quick to dismiss that they don’t have that [bias] because then you haven’t really reflected on it and I bet that you do. So it makes me uncomfortable when people are like “oh*,* but I’m not racist” then like*,* I’m not saying that. So*,* unless you’re super aware of these things that it means that you’re more… I don’t know*,* you’re at risk of falling into those things even if you don’t mean to. So these women feel like they’re not listened to*,* they feel like their pain isn’t taken seriously. They feel like they’re not valued*,* if they complain*,* they’re seen as being a nuisance rather than having like an ethical*,* moral right to complain because they are a person*,* a valid person in themselves that’s had a wrong happened to them. And then you’ve got all of the social stuff*,* we live in a country that’s weirdly right wing at the moment and there’s huge levels of deprivation and literally generation levels of prejudice and discrimination. So all of that is kind of maternity care*,* can be a sort of a microcosm for that as well”. P12*,* Midwife.*

Some universities were considered to be leading the way in training on biases and stereotypes in maternity care. However, student midwives reported tension between theory and practice, encountering inappropriate comments and behaviours towards vulnerable women in labour. These encounters represent an issue for students, who may not feel able to challenge members of staff or escalate non-clinical concerns due to fear of retaliation and the difficulty to measure and proof biases.

*“I’ve heard comments from midwives like “oh*,* you walk in the room and you can tell that she’s got a safeguarding” […] or if this is like her 9th baby*,* some people are like “oh*,* has she got any safeguarding?” […] or you hear things like*,* “oh*,* that was good for an Indian lady or she did well for an Indian lady”*,* […] “oh*,* she’s Indian*,* she might get an epidural” […] You don’t see someone go “Oh*,* she’s white British*,* she’s going to have an epidural”. What’s the reason that you’re gonna point out that she’s Indian? […] Is that improving her care by you saying that? No […]. So yeah*,* I can definitely see how they’ve got poorer experiences”. P9*,* Student Midwife.*

*“I feel more comfortable escalating something like that [obstructed labour] because it’s clinical and it’s measurable and the potential of ignoring that is catastrophic […] I would find harder to deal with [racism and stereotyping] because typically*,* […] people are very quick to kind of make light of it*,* “No*,* I didn’t mean it like that*,* […] don’t take it seriously*,* don’t make a big fuss” […]. But yeah*,* university do tell us that we escalate these things*,* but when you’re being supervised by people who are signing off your competences […] they could potentially decide to put me on a progression plan […]*,* it’s actually really hard to […] change the perceptions that everybody then develops about you…”. P10*,* Student Midwife.*

## Discussion

This is the first qualitative study to explore whether and how the socio-technical context of EFM management can manifest in care disparities. The findings illustrate key ways in which the intersectional dynamics between women and clinicians may have influenced clinical practices surrounding EFM in labour. In contrast to its characterisation as a neutral objective metric, EFM-informed decision-making and associated collaborative care practices are deeply embedded within the socio-technical fabric of the labour ward [50]. While factors such as ethnicity, SES, language proficiency, cultural beliefs and health literacy are not explicitly acknowledged in EFM interpretation, they become manifest in the broader clinical context and decision-making processes. Clinicians may have differentially escalated concerns, with thresholds for intervention being unconsciously adjusted according to ethnicity, SDH and maternal preferences– highlighting the potential inclusion of personal and subjective biases in risk assessment processes. The use of race or ethnicity alone to evaluate physiological differences in heart rate patterns can exacerbate perinatal health disparities by accepting that race and ethnicity are biological constructs as opposed to conforming with a socio-political classification [21]. Aquino and colleagues [51] undertook a descriptive cross-sectional study to assess potential differences in heart rate patterns according to maternal ethnicity. Despite the lack of statistical significance in their results, the authors highlighted a parameter that showed to be different among Black women compared to other ethnic groups. Using race and/or ethnicity to explain potential biological differences and health disparities without acknowledging biases could result in racial bias and stereotyping, which could lead to higher clinical suspicion and potentially pathologisation of Black and Brown skin women [52]. In our study, using ethnicity to consciously or unconsciously inform EFM management could have been influenced by media, reports and evidence to date on ethnic minoritised women and disproportionate perinatal adverse outcomes. On the other hand, screening, acknowledging and addressing SDH to improve perinatal outcomes and maternity care is paramount [53]. Nevertheless, implementation in clinical practice proves to be difficult due to the limited evidence available to disentangle the complexity of the SDH net [54], the lack of evidence to support actionable solutions to address SDH, the effectiveness of clinical interventions, and the perception of SDH falling off health professional’s remit [55]. Addressing SDH removes the burden off women and focuses on broader determinants that link to inequalities [54].

Personalised care and support plans in maternity, central to Better Births [56] and a cornerstone to the NHS Long Term Plan [57], are known to have a positive impact on health inequalities. NHS England expects maternity staff to complete cultural competence and unconscious bias training [58] in response to the “constellation of biases” that prevent women with complex and diverse intersectional factors from receiving equitable maternity care [59]. Nevertheless, there is a lack of standards about auditing, supporting and financing cultural competence and unconscious bias recommendations, and it is up to individual organisations whether they implement them and how [60]. Several participants reported a lack of cultural competence and unconscious bias training in their work place. Also, some study participants reported practising with a “SDH and colour blindness” lenses, which illustrates a socially and politically correct narrative to portrait a distorted equality. Colour blindness and, by extension, SDH blindness, represent modern racism [61, 62], where the blame is placed on the individual and their life choices [62, 63]. This blindness also fails to acknowledge the role of system-level SDH, such as structural and institutional factors, and its impact on care provision and health outcomes. Moreover, this blindness reduces sensitivity to bias and racism, and negates the opportunity for addressing it [64]. Addressing SDH and unbiased, culturally competent labour care is vital for ensuring equitable and truly personalised care for women and their babies. It is also an ethical, human rights and social justice matter, to ensure that every woman and baby can achieve their highest health possible and free from harm [65]. A SDH approach to the provision of EFM care could address existing disparities and improve care by fostering empathetic and humanised relationships with women after self-reflecting on existing structural bias and barriers that lead to suboptimal outcomes [66]. Although some training initiatives have been piloted [67, 68], further work is needed to better understand approaches to influence healthcare providers and staff to address racial health inequalities.

In our study, we noted EFM practices impacted by the intersection between maternal SDH and professionals’ preferences. Similarly, previous research has also concluded that labour ward professionals’ personal beliefs influenced interpretation and decision making in CTG practice [69]. Moreover, willingness to intervene during labour is linked to risk perception, years of experience and number of hours worked [70]. Decision-making styles are also influenced by personal and professional attributes [71], while midwives’ decision making in particular is influenced by attitudes on physiology, woman-centredness, shared decision-making, collaboration with other professionals [72] and prior exposure to trauma [73].

Access to and use of interpreting and translation services varied across participants’ reports, with a generalised limited use of such services throughout the labour process. Access and quality of interpreting services is a recurrent theme in patient safety incidents in the UK and an important factor that negatively influences women’s experiences of care [74]. Furthermore, evidence to date around communication needs in maternity care have also identified disrespect, discrimination and stereotyping [75, 76], as well as assumptions around symptoms made on the basis of language ability and/or ethnic group [19]. Communication barriers also evidenced concerns around poor risk communication, limited choices, pressure to make decisions [77], informed consent, misunderstandings and inability to articulate needs, limiting women’s involvement in decision-making processes [78]. Echoing existing evidence on interpreting services in the NHS, we found issues around access and use of these services during labour, with concerns around informed consent practices, choices, and regular communication [79]. Therefore, the perceived delegation of responsibility during labour identified in this study could be explained by multiple barriers, such as communication barriers, unawareness of the maternity system, challenges in accessing and navigating the NHS and the intersection of multiple complex risk factors [80]. Language barriers and differing levels of health literacy create significant challenges in managing EFM findings and shared decision-making. While there is an ideal of all women having access to professional translation services during labour to provide “safe, consensual and personalised care” [81], achieving this in the context of the labour ward proves difficult. Often, translation and interpreting services are being used selectively or replaced by family members or untrained individuals, leading to miscommunications and incomplete information transfer needed for shared decision-making and woman-led care advocacy [82]. Clinicians may deliberately or inadvertently excluded women who struggle with language and literacy, contributing to a perpetual cycle of care that is less collaborative and more prone to undermining trust and systemic disparities of care, particularly around informed consent practices, choices, and regular communication [79]. The current obstetric model prioritises clinician’s recommendations by reinforcing a hierarchical system [83, 84], which is particularly evident in disadvantaged women.

Given the inherent ambiguity and interpretive flexibility of EFM traces, clinicians may selectively interpret EFM data to rationalise a course of action that aligns with their own clinical preferences rather than following a collaborative process. EFM, then, becomes a tool through which clinicians can assert control, potentially overriding the woman’s autonomy. EFM has been described as “symbolically removing the fetus from the context of the mother-placenta and representing it in the form of its heartbeat on the screen”, relegating women as mere containers [85]. Anthropologist Davis-Floyd pictured an illusion where EFM devices appeared to “keeping the baby’s heart beating”. In this illusion, clinicians felt that disconnecting the woman from the fetal monitor “will cause the baby’s heart to stop” [86]. Moreover, the use of EFM physically controls the woman’s body to produce a good fetal heart recording, “externalises the fetus” and allows clinicians to intervene to save the unborn baby [85]. This “fetal centring”, supported by fetal surveillance, among other interventions, has been found to lead to “coercive and restrictive practices intended to dissuade women from making choices that might present a risk to the fetus” [90]. Moreover, EFM practices often lack informed consent, as fetal monitoring is considered part of the standard package of labour care that comes with routine labour interventions [87]. MacLellan and colleagues [88] concluded that women were unable to make informed decisions around fetal monitoring during labour due to limited discussions during the antenatal period. Furthermore, health professionals often become accustomed to EFM and obviate IA for low risk women [88]. Some women have also reported feeling disrespected when declining EFM, while other did not think they had a choice [89] Consequently, contradictory forces then arise on the labour ward: women-centred and autonomy vs. fetal centring care [90], where EFM has increasingly gained a “clinical decider” role [91]. Also, EFM coexists with organisational factors- medical hierarchical systems, high workload, language barriers, recent adverse events [92], staffing shortages and fear of litigation [50], which reinforce the overreliance on EFM [93]. Such organisational factors often undermine women’s autonomy and involvement in decision-making [77, 92].

A lack of involvement in decision-making processes contributes to disparities in care provision, health outcomes and experiences of care [50]. In line with our findings, examples of this lack of autonomy and involvement in decision-making was identified around the provision of pain relief [94] and choice of place of birth [14]. In occasions, limited access to choices in labour care becomes influenced by maternal ethnicity, suggesting a disproportionate categorization of ethnic minoritised women as high risk, requiring obstetric care during labour, and leading to iatrogenic harm [95]. Moreover, tensions can arise that jeopardise woman’s autonomy when women’s preferences and clinician’s recommendations clash [50], particularly around cultural practices, ignoring midwifery principles of women-centred care [96] and neglecting women’s needs [89]. We identified how cultural and social backgrounds influence expectations of maternal agency during labour, leading to potential disparities in how care was enacted. More specifically, women from certain cultural backgrounds, especially those from non-European countries, were seen as more likely to internalise beliefs that provide protection in a patriarchal culture, i.e. showing a tendency to defer decision-making to clinicians. This deference is intertwined with cultural norms that value trust in expert knowledge and accountability for the baby’s wellbeing. In line with our findings, researchers have found that ethnic minoritised and culturally marginalised women find themselves “working from a disadvantaged point” and having to “earn their right to be heard”, which often relegates them to a passive role in their care [77]. This lack of agency was also evident in a nationwide study of Black women’s experiences of maternity services in the UK [97]. By contrast, well-educated women and those more familiar with the healthcare system were perceived to exhibit much greater agency, being more vocal, asking more questions, and insisting on a more collaborative approach. Similarly, biases were witnessed in the form of greater care and patient advocacy afforded to privileged, White professional, socio-cultural normative women. This “VIP syndrome” has been documented in other clinical areas [98] and translates into providing faster access to care, more in-depth discussions and leading care.

Participants reported examples of clustering women’s labour behaviours, attitudes, preferences and needs according to shared ethnic and cultural characteristics. Stereotypes and biases may have influenced the EFM management and care provided to women. In order to promote equity in clinical decision-making and care provision, it is imperative to dismantle race-based medicine and replace it with a race-aware approach that considers social and environmental factors such as structural racism and SDH [99].

Regarding the current midwifery and NHS crisis, labour and birth are areas suffering the effects of understaffing, which leads to reduced choices and control on the birth place, affecting the standards and quality of care. Chronic understaffing and heavy workloads also leave clinicians exhausted, without breaks, burned out and dealing with highly stressful situations. The current crisis is encouraging a ‘conveyor belt’ style of labour care [100], as well as fast, automatic and biased thinking [101]. Furthermore, recent high profile legal cases, investigations and events have fuelled a risk-aversion culture, over-medicalisation and defensive practice to avoid litigation and professional consequences [102], which could explain a different threshold for escalation and action in some vulnerable women. Midwives in our study reported feelings of dissatisfaction as a result of being caught in a system that promotes such “conveyor belt” style to labour care. Similarly, Westergren and colleagues [103] noted that the organisation of labour wards are “hierarchical and based on traditional masculine values, such as rationality, efficiency and productivity”. Midwives become task-oriented, leaving the essence of being with women aside, which increases birth interventions [103]. Echoing our participants’ sentiment of “over CTG-ing women”, Quattri [102] also observed an overuse of EFM that led to unnecessary interventions. This overuse of EFM highlights a tension between guidelines compliance and unnecessary interventions that could increase women’s risk. Such defensiveness in practice has been identified as a contributor to health inequalities for underserved populations [104]. Moreover, recent evidence also found that staff workload was most frequent in cases of high-level obstetric clinical incidents amongst women from ethnic minoritised groups [105]. Therefore, current maternity systems fail to acknowledge the paradox of iatrogenic harm caused by risk mitigation interventions, such as EFM practices, as well as women’s social needs that require additional resources and time allocations. As Sudhinaraset and colleagues stated, reproductive autonomy can only be achieved by providing access to culturally and linguistically appropriate maternity care [106].

In bringing these seven themes together, this study has evidenced how the interpretation and use of EFM is not just a concern with technology and its data, but is deeply entangled with people’s interactions, SDH and organisational factors. Clinical teams have to operate in a complex socio-technical environment where unconscious biases, cultural expectations, communication challenges, time pressures and resource constraints mediate the objectivity of EFM readings. Acknowledging and addressing intersectional dynamics is a key concern for ensuring equitable health outcomes and personalised care [53]. However, its implementation in clinical practice is challenging because of the nuanced complexity of the SDH net [54].

Findings from this study show necessary practice and policy implications focusing on access to culturally appropriate translator services and universal provision of culturally and linguistically appropriate care for all women. Intersectional clinical care to understanding different levels of vulnerability and care needs of women with multiple intersecting identities is crucial to achieving equitable care, outcomes and reproductive autonomy [107]. Moreover, it is fundamental to dismantling race-based medicine by following a race-aware approach to clinical decision support that encompasses structural racism and SDH [99]. Clinicians must be given time and appropriate resources to learn and reflect on biases when looking after women from diverse backgrounds and with different care needs. Maternity services need to invest in decolonisation strategies to challenge current structures and practices that mainly cater for normative conforming pregnant women [108]. It is also important to regularly undertake health equity audits to evaluating access to and use of culturally and linguistically appropriate care, choices, informed decisions and informed consent regarding labour interventions, such as fetal monitoring. Moreover, all women should receive information about fetal monitoring practices during pregnancy to support informed decisions. Future research should address how women with multiple intersectional identities navigate EFM care and their experience of involvement in decision-making processes. Research efforts should also address the role of SDH in labour outcomes and the potential for tailored care pathways.

In order to produce transformative change, maternity services should strive to co-designing and co-producing maternity care policy, practice and service delivery [109] with women from ethnic minoritised groups, socio-economic disadvantaged backgrounds and those who experience communication barriers. Also, it is paramount to introduce institutional reflexivity and accountability approaches that involve self and institutional assessment and evaluation of power [110]. In broader terms, policy makers and public health agencies should address the lack of national standards for risk assessing pregnant women in the UK supported by an intersectional approach.

### Strengths and limitations

A key strength of this study was the use of vignettes and open-ended questions to encourage participant’s reflections on a sensitive topic. On the other hand, we acknowledge serveral limitations. The majority of the participants interviewed were White, which may have influenced their perspectives on biases and disparities in labour care and electronic fetal monitoring. The limited diversity of the sample could increases the potential for the Black, migrant, and disadvantaged women being framed as “the other”. It is important to engage with a representative selection of clinicians to explore their experiences and to avoid normalising and increase bias. Future research should address the limitation of representation to capture diverse voices. Although we included a fair representation of clinicians, despite targeted efforts, we did not receive many expressions of interest from obstetricians. Therefore, this study has mainly captured the midwives’ perspectives. Future research studies should identify strategies to recruit obstetricians and explore their views on the topic.Moreover, participants’ views and sentiments on maternity care disparities and issues in the current provision of maternity care in the NHS were fairly heterogeneous. Therefore, future studies should pay attention to capturing divergent voices on the topic.

We also acknowledge the limitations of discussing past events and sensitive topics, and the possibility of including a degree of recall and social desirability bias. Another limitation was the inclusion of clinicians only. Therefore, future research could look at undertaking a mixed methods approach, including the experience of women with different intersectionalities and clinical outcomes data on EFM to provide a full view on the subject. 

## Conclusion

EFM management cannot be understood from an individualistic perspective where clinicians interpret and escalate findings around fetal wellbeing in labour. EFM is a tool that is socially and contextually interpreted and subjected to systematic contextual influences, maternal SDH and biases that could potentially contribute to disparities in labour care and outcomes. A socio-technical systems approach that acknowledges the intersectional dynamics between clinicians, pregnant women and systemic and structural factors is imperative to drive equity in EFM and labour care.

## Data Availability

The data that support the findings of this study are not openly available due to reasons of sensitivity and are available from the corresponding author upon reasonable request.

## Abbreviations

CDST: Clinical Decision Support Tools
CTG: Cardiotocography
EFM: Electronic Fetal Monitoring
IA: Intermittent Auscultation
PPIE: Patient and Public Involvement and Engagement
RTA: Reflexive Thematic Analysis
SDH: Social Determinants of Health
SES: Socioeconomic Status
UK: United Kingdom

## Footnotes

1 EFM management and EFM care are used interchangeably throughout the paper to refer to the clinical practices involved as a result of using EFM during labour, i.e. interpretation, escalation, communication and action.

2 *Ethnicity and race: the working definition in the paper is that race and ethnicity are socially constructed concepts that refer to one’s cultural and social identity as opposed to biological referents* [21].

3 *The term Social Determinants of Health refers to “conditions in which people are born*,* grow*,* work*,* live*,* and age*,* and the wider set of forces and systems shaping the conditions of daily life”*,* i.e. “the non-medical factors that influence health outcomes”* [22].

4 *Health and care disparities refer to preventable differences in health outcomes*,* access to care*,* and the quality of healthcare services between population groups defined by age*,* race*,* ethnicity*,* socioeconomic status and geographic location. These disparities manifest in higher rates of maternal morbidity and mortality and worse care experiences. It also highlights that “practices and policies are impacted by interpersonal and structural bias*,* such as social*,* structural*,* and political determinants of health impacting the antenatal*,* intrapartum and postpartum periods”* [29].

5 In this paper, women of colour and minoritised ethnic women refer to the same concept. “Minoritised ethnic” is a recommended term that reflects the power-dynamics within society beyond mere statistics [35].

6 *Cardiotocography (CTG) and EFM are used to refer to the same concept*,* i.e. fetal heart monitoring.*
